# Effect of Docosahexaenoic Acid and Eicosapentaenoic Acid Supplementation on Sleep Quality in Healthy Subjects: A Randomized, Double-Blinded, Placebo-Controlled Trial

**DOI:** 10.3390/nu14194136

**Published:** 2022-10-05

**Authors:** Kaori Yokoi-Shimizu, Kenichi Yanagimoto, Kohsuke Hayamizu

**Affiliations:** 1 Food Function R&D Center, Nippon Suisan Kaisha, Ltd., Tokyo 192-0919, Japan; 2 Laboratory of Food Chemistry, Yokohama University of Pharmacy, Yokohama 245-0066, Japan

**Keywords:** omega-3, docosahexaenoic acid, eicosapentaenoic acid, sleep, unsaturated fatty acid, middle- and older-aged, healthy subjects

## Abstract

Docosahexaenoic acid (DHA) and eicosapentaenoic acid (EPA)—omega-3 fatty acids with various functions—influence sleep in children and young adults. However, only limited studies on their effects on sleep in middle- and old-aged adults have been reported. Therefore, we investigated the effects of DHA and EPA on sleep quality in subjects aged ≥ 45 years. We performed a randomized, placebo-controlled, double-blinded, parallel-grouped study, in which we randomly assigned 66 healthy Japanese males and females. Each individual received six 480 mg capsules containing 576 mg DHA and 284 mg EPA per day (DHA/EPA group, *n* = 33), or corn oil (placebo group, *n* = 33), for 12 weeks. Before and after the intervention, the Oguri-Shirakawa-Azumi sleep inventory MA version (OSA-MA) and the sleep state test were conducted. In the DHA/EPA group, factor III (frequent dreaming) scores among the OSA-MA scores were significantly improved compared to the placebo group. Additionally, sleep state tests revealed that sleep efficiency improved in the DHA/EPA group. To our knowledge, this study is the first to report that DHA/EPA improves sleep quality in middle- and old-aged individuals, even at doses lower than those administered in previous studies.

## 1. Introduction

Sleep quality is evaluated based not only on sleep duration, but also on multiple aspects, such as the state of sleep–wake rhythm, and is an important component for a healthy lifestyle [1,2]. In recent years, numerous studies on sleep have reported that a decline in sleep quality is closely related to the progression of lifestyle-related diseases, such as diabetes, hypertension, ischemic heart disease, and depression [3,4,5,6,7,8,9].

In other words, considering strategies to improve the quality of sleep is one of the most important issues in maintaining health or preventing a decline in the quality of life. With the advancement in sleep research worldwide, sleep problems are being recognized as an important issue and are being considered as one of the national health strategies in many countries [10,11]. For example, the United States has established “Sleep Health” under the agenda “Healthy People 2020”, and is working on promoting sleep health by setting specific targets and measures [10]. In Japan, the Ministry of Health, Labour and Welfare has also formulated “Sleep Guidelines for Health Promotion 2014” to raise awareness of the importance of getting proper sleep [11]. Sleep quality is greatly influenced by melatonin, which has a regulatory effect on circadian rhythms, including the rhythms of sleep, wakefulness, and hormone secretion [12,13], and is known to decrease with age [14,15]. Studies on improving the quality of sleep for middle- and old-aged adults, who are prone to decreased sleep quality, are expected to provide solutions for important public issues.

Docosahexaenoic acid (DHA) and eicosapentaenoic acid (EPA) are unsaturated fatty acids extracted and refined from fish, and they cannot be synthesized by the human body. Their pharmacological effects have mainly been reported in terms of their ability to decrease blood lipid levels, reduce the risk of developing cardiovascular disease, and improve brain function [16,17,18,19,20,21,22,23]. Given that DHA/EPA exists as a constituent lipid of all cell membranes [24,25,26,27] in the body and its metabolites are thought to have various physiological activities [28,29,30], DHA/EPA can provide numerous benefits. Moreover, DHA and EPA have been reported to alter the membrane phospholipid composition of the pineal gland, which produces melatonin and modulates melatonin production [31,32], and thus are considered to influence sleep.

The correlation between seafood, especially its DHA/EPA content, and sleep quality has been previously demonstrated. For instance, in an epidemiological study, Del et al. [33] reported that adult individuals with good sleep quality had a high intake of oily fish; additionally, Katagiri et al. [34] reported that poor sleep quality was associated with low fish intake. Later, Judge et al. [35] and Michael et al. [36] conducted intervention studies on DHA/EPA intake in children and adolescents. However, only a few intervention studies have been conducted on middle- and old-aged adults with poor sleep quality [11]. Given that DHA and EPA are recognized worldwide for their ability to reduce the risk of heart disease, improve blood lipid levels, and enhance cognitive function—all of which decline with age—we hypothesize that DHA/EPA may also have effects on sleep in middle- and old-aged people. Therefore, in this study, we aimed to determine the effects of DHA/EPA on sleep quality in middle- and old-aged subjects.

## 2. Materials and Methods

### 2.1. Study Design

This study used a randomized, placebo-controlled, double-blinded, and parallel-grouped design. The participants were randomly assigned to receive DHA/EPA or placebo for 12 weeks.

### 2.2. Ethics Statements

The study complied with the Declaration of Helsinki (2013) and the Ethical Guidelines for Medical Research Involving Human Subjects. The study was approved by the Ethics Committee of the Takara Clinic, Seishinkai Medical Corporation (Shinagawa, Tokyo, Japan) (approval code: 2009-00214-0015-16-TC) and University Hospital Medical Information Network Clinical Trials Registry: UMIN000041864.

### 2.3. Participants

**Figure 1:**
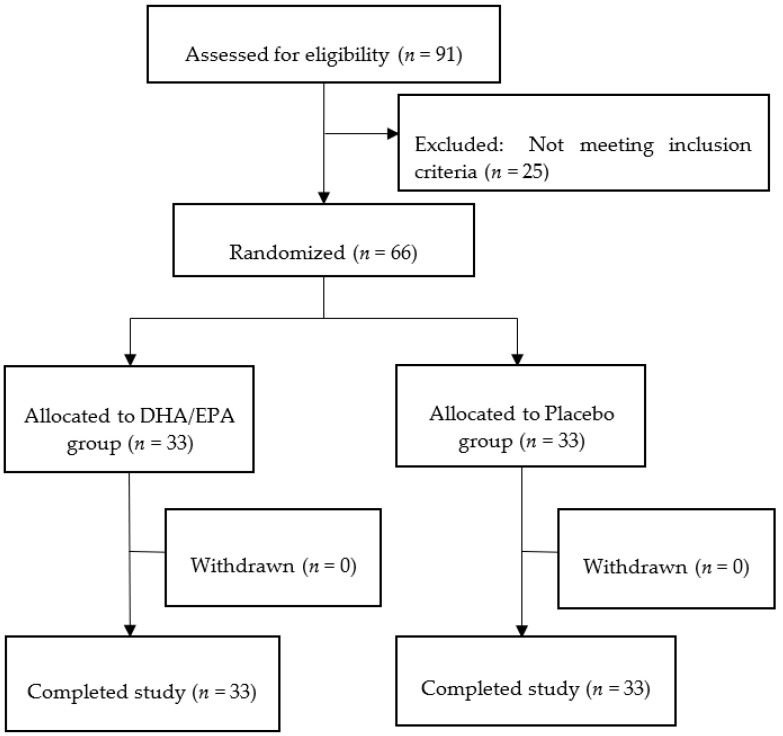
Participant flow diagram.

The participants of the study were healthy Japanese adults aged ≥ 45 years with poor sleep quality. The exclusion criteria were as follows: (1) undergoing medical treatment or having a medical history of malignant tumors, heart failure, or myocardial infarction, and having a pacemaker or an implantable cardioverter defibrillator; (2) undergoing treatment for cardiac arrhythmia, liver disorders, kidney disorders, cerebrovascular disorders, rheumatism, diabetes mellitus, dyslipidemia, hypertension, or any other chronic diseases; (3) the presence of factors that affect their sleep due to their home environment (e.g., living with an infant under 1 year of age or a person requiring nursing care, or sleeping with more than one person in one bed); (4) having irregular sleep habits due to irregular lifestyles (irregular meal timing and insufficient sleep) or night shifts; (5) excessive drinking (average of more than about 20 g/day in absolute alcohol intake); (6) undergoing treatment for sleeplessness or sleep disorders; (7) consuming oily fish four or more times a week; (8) intake of any functional food or supplements that could influence the outcome of the study; (9) pregnancy or lactation; (10) having allergies, especially to fish or gelatin. The study participants were recruited through a recruitment websites for monitoring. Of the 91 potential subjects, 66 were included in this study (Figure 1). The 91 applicants underwent the Oguri-Shirakawa-Azumi sleep inventory MA version (OSA-MA) test [37] at the point of screening. In addition, the Profile of Mood States 2nd Edition—Japanese version (POMS2) [38,39] and the sleep state test (sleep scan SL-511-WF2, Tanita Co., Ltd., Tokyo, Japan) [40,41] were performed. The study physicians performed face-to-face interviews and blood and urine tests at baseline and at week 12. This study was conducted at the Medical Corporation Seishinkai, Takara Clinic (Shinagawa, Tokyo), and the Nerima Medical Association Minami-machi Clinic (Nerima, Tokyo). All participants enrolled in this study provided written informed consent after receiving an explanation of the study procedures, which were carried out in accordance with the Helsinki Declaration.

### 2.4. Randomization and Blinding

The allocation manager assigned all participants after reaching the target number of subjects, generating random numbers on a computer, and creating an allocation table using a completely random method with predetermined variables as factors; participants were randomly allocated to either the DHA/EPA supplementation group or the placebo group based on the allocation table. The following variables were equally distributed in both groups: sex, age, and the OSA-MA score. The prepared allocation table was provided only to the person in charge of shipping the test foods, and the test foods were mailed to the participants according to the allocation table. Randomization and allocation data were concealed from the researchers, clinicians at the medical institutions, staff members of the study institution, members of the ethics committee, and the clinical laboratories and participants until the final analyses were completed. The allocation table was sealed and stored until it was opened by an independent controller.

### 2.5. Treatment

During the treatment, the participants had no health problems or allergic reactions. Participants took either six 480 mg capsules of DHA/EPA containing refined fish oil (DHA: 576 mg; EPA: 284 mg) per day or a placebo, which contained corn oil without DHA and EPA, for 12 weeks. The placebo and treatment capsules were identical in size and shape and similar in appearance. Participants were instructed to not change their daily habits, such as the duration of sleep, and the quantity of alcohol and fish intake. The participants did not drink alcohol before the sleep quality test and did not consume anything except water 10 h before blood sampling.

### 2.6. Subjective Evaluation

#### 2.6.1. OSA-MA

Participants underwent the OSA-MA test at home once daily for three consecutive days before the baseline evaluation date and during week 12 before the evaluation test was conducted, immediately after waking. The final score used was the average of the three days’ scores.

The OSA-MA test included 16 questions, and the results were consolidated into 5 factors: (1) sleepiness upon waking, (2) initiation and maintenance of sleep, (3) frequent dreaming, (4) refreshing on rising, and (5) sleep length.

#### 2.6.2. POMS2

The participants completed POMS2 at baseline and during week 12 of the evaluation test. POMS2 included 35 questions, and the results were consolidated into eight factors: total mood disturbance, tension–anxiety (TA), depression–dejection, anger–hostility, vigor–activity (VA), fatigue–inertia, confusion–bewilderment, and friendliness.

### 2.7. Objective Evaluation

#### Sleep State Test

The sleep state was monitored using the sleep scanner SL-511-WF2 (Tanita), a mat-type monitor that evaluates the sleep state by collecting breath, pulse, and body movement data from the subjects. Participants underwent a sleep quality test while sleeping for seven days before the baseline and week 12 evaluation tests. The score used was the average of the seven days’ scores. Sleep state tests can be evaluated as follows: sleep efficiency (the ratio of actual sleep time to total sleep time), sleep latency (the difference, in minutes, between in-bed time and sleep onset), total sleep time (the total number of minutes scored as “asleep”), actual sleep time (total sleep time minus average awaking length), average awakening length (the average length, in minutes, of all awakening episodes), and an average ratio of awakening (calculated as time of awakening length/total sleeping time × 100).

### 2.8. Blood Sampling

Plasma polyunsaturated fatty acid (arachidonic acid, EPA, and DHA) composition was measured at baseline and during week 12 by the LSI Medience Corporation (Tokyo, Japan).

### 2.9. Dietary Survey

Participants were examined via a dietary survey seven days before the baseline and week 12 evaluation tests using a Calorie and Nutrition Diary [42]. The average of the seven days’ scores was used to evaluate energy, protein, fat, carbohydrate, omega-3 fatty acids, DHA, and EPA levels.

### 2.10. Safety Evaluation

At baseline and week 12, blood samples were collected under 10 h fasting conditions. The following blood parameters were measured: white blood cell count, red blood cell count, hemoglobin level, hematocrit, platelet count, and total protein, albumin, aspartate aminotransferase, alanine aminotransferase, lactate dehydrogenase, total bilirubin, alkaline phosphatase, γ-glutamyl transpeptidase, urea nitrogen, creatinine, uric acid, sodium, chlorine, potassium, total cholesterol, LDL cholesterol, HDL cholesterol, triglyceride, fasting plasma glucose and glycated hemoglobin (HbA1c) levels. Urine samples were obtained at baseline and week 12, and the following urine parameters were measured: protein, glucose, and occult blood levels. All plasma biochemical variables were measured by the LSI Medience Corporation. Systolic and diastolic blood pressure, pulse rate, body height and weight, and body mass index (BMI) were measured at baseline and week 12.

### 2.11. Sample Size

The sample size was calculated using the Montgomery study [43], which evaluated the effect of DHA on sleep quality using a sleep meter. We used *d* = 0.8 based on Cohen’s study [44], assuming that the difference in scores between the test group and placebo group was large. Setting α = 0.05 and 1-β = 0.80, we calculated the required sample size to be 62 (31 in each group). We recounted the power (1-β), and the score obtained was 0.83.

### 2.12. Statistical Analysis

The data have been presented as means and standard deviations and compared between groups using analysis of covariance (ANCOVA), with the baseline as the covariate. Participants’ backgrounds were compared between groups using the Student’s *t*-test or the chi-square test. All statistical analyses were performed using two-sided tests, and the significance level was set at 5%. IBM SPSS statistics software (version 23 and higher; IBM Corp., Armonk, NY, USA) was used, with other statistical software used as needed.

## 3. Results

### 3.1. Participants

As shown in Figure 1, of the 91 potential subjects, 66 met the criteria and were randomly assigned to either group. All enrolled subjects completed the study in accordance with the ethical compliances. Each group comprised 33 patients (male: 11, female: 22). The subjects’ background information is presented in Table 1.

In the DHA/EPA group, the serum concentrations of DHA and EPA were significantly higher than those in the placebo group (Table 2). No items in the dietary survey were significantly different between the two groups (Table 3).

### 3.2. Subjective Evaluation

The average of each score is presented in Table 4. In the DHA/EPA group, the post-ingestion score of factor III (frequent dreaming) was significantly higher than that in the placebo group (*p* = 0.043). Regarding POMS2, no items were significantly different between the two groups (Table 5).

### 3.3. Objective Evaluation

Each score on the sleep state test, mean, SD, and the statistical results are shown in Table 6. In the DHA/EPA group, the post-intervention sleep efficiency score was significantly higher than that in the placebo group. Two participants (one in the DHA/EPA group and the other in the placebo group) did not undergo the sleep state test during the post-ingestion period; hence, their score was treated as a missing score.

### 3.4. Adverse Events

Under the conditions of this study, no adverse events, including in the blood and urine test results, were identified during the study period. The study physician judged that all conditions were unrelated to food testing. Supplementary Table S1 shows the results of the blood and urine tests.

## 4. Discussion

The present study confirms the improvement in sleep quality via the continuous intake of DHA/EPA for 12 weeks in subjects over 45 years of age, who are generally considered to have poor sleep quality [14,15].

Previous studies on DHA/EPA and sleep quality included individuals of various age groups and reported consistent efficacy in infants [35,45] and children (4–18 years) [43,46]. A meta-analysis conducted in 2019 confirmed the efficacy [47]. However, few intervention studies have been reported in adults, and only Hansen et al. [48] and Michael et al. [36] evaluated sleep, including objective measures such as sleep status, outside of this study.

Hansen et al. [48] showed that a fish diet (Atlantic salmon) had beneficial effects on sleep in 95 male sex offenders (21–60 years old, mean: 42 years). Michael et al. [36] reported increased sleep efficiency and improved subjective evaluation following DHA/EPA intake in adults (25–49 years old, mean age in the placebo group—36.89 years; in the DHA group—37.41 years, and the EPA group—35.89 years). To date, no studies have confirmed the effects of DHA/EPA on sleep quality in middle-aged males and females with low sleep quality. In a study that did not primarily evaluate sleep, Watanabe et al. [49] found validity at some measurement points in the subjective assessment of sleep in 80 female nurses (20–59 years, mean age: 30.5 ± 7.8 years). No effect of DHA/EPA on sleep was found in pregnant women (18–35 years; doses: 220 and 300 mg DHA/day) [50,51] and in females with menopausal symptoms (40–61 years; dose: 525 mg EPA+DHA/day) [52].

Hansen et al. [48] studied the effects of 4.8 g EPA+DHA/day in 300 g of Atlantic salmon, and Watanabe et al. [49] studied the effects of 2000 mg of DHA+EPA per day, on sleep. Michael et al. [36] evaluated the effects of high DHA (1170 mg/day of DHA+EPA) and high EPA (1260 mg/day of DHA+EPA), and found increased sleep efficiency and improved subjective evaluation. Thus, in this study, we set the daily intake of DHA+EPA at 860 mg/day, considering it as the minimum intake quantity that influences sleep, as no effect was observed at lower doses. The results of this study are significant for reducing the burden of DHA/EPA intake for those with sleep issues and improving their quality of sleep, thereby contributing to improving their QOL. In contrast, the subjects in the study who showed no effect were pregnant women and subjects with diseases; it is possible that these subjects require higher doses of DHA/EPA than healthy subjects do. In a cohort study on DHA/EPA and sleep [53], it was reported that in patients with obstructive sleep apnea (OSA), a sleep disorder disease, blood DHA/EPA levels were lower in healthy subjects or patients with mild symptoms than in those with severe symptoms. One of the major functions of DHA/EPA is to improve brain function. Lower blood DHA/EPA concentrations have been reported in patients with dementia and depression than in healthy subjects [54,55]. These findings suggest that higher levels of DHA/EPA may be required for patients with disorders than for healthy patients. Nevertheless, the effects of low doses of DHA/EPA on sleep may require further evaluation in the future.

In this study, the OSA-MA score was significantly higher in only factor Ⅲ (frequent dreaming) after 12 weeks in the DHA/EPA group than in the placebo group (Table 3). “Frequent dreaming” was assessed by the questions “I had many nightmares” and “I dreamed often” [37,56]. Although only “frequent dreaming” was validated in this study for the subjective evaluation of OSA, each variable, such as “frequent dreaming”, is an independent indicator. As stated in “How to use the MA version of the OSA Sleep Questionnaire” [56], “frequent dreaming” is particularly affected by stress. Sleep and stress are known to influence each other closely, and various types of stress reduce sleep quality [57,58,59,60,61]. Epidemiological studies on stress and sleep have reported a higher prevalence of sleep problems in individuals with high subjective stress, tension, and anxiety [62,63]. To examine the effects of stress on sleep, a stratified analysis was performed based on the different stress states of the subjects before the start of the study. In the group with TA higher than the within-subject average (*n* = 27), “frequent dreaming” was significantly improved in the DHA/EPA group compared with the placebo group after 12 weeks of intake (pre-intake DHA/EPA group—23.0 ± 7.2, placebo group—16.9 ± 7.8; post-intake DHA/EPA group—24.5 ± 4.3 *, placebo group—18.5 ± 6.7; * *p* < 0.05); post-intake measurements were compared between groups using ANCOVA with pre-intake measurements, with covariates and groups as factors. In contrast, a stratified analysis was performed for those TA below the within-subject average; however, no significant differences were found between groups. These results indicate that DHA/EPA intake improves “frequent dreaming”, particularly in stressed populations. Since “frequent dreaming” is particularly influenced by stress, as mentioned earlier, we consider that only the OSA-MA score of “frequent dreaming” improved.

The study has confirmed that sleep efficiency after DHA/EPA administration was significantly higher than that after placebo administration. As mentioned previously, the correlation between sleep quality, stress, and sleep efficiency normally declines under certain stressors. In this study, the subjective evaluation in POMS generally showed improvement before and after intake due to the placebo effect [64,65,66], but there was an increase before and after intake for the questions “I feel depressed”, “I feel upset”, “I feel lonely”, “I want to fight easily”, “I cannot do anything by myself”, and “I am at a loss” (data not shown). Therefore, sleep efficiency could be maintained by DHA/EPA intake in the study participants experiencing stress. There are many possible stressful situations in daily life, and one possible stressful factor during the study period was the spread of COVID-19.

In this study, a sleep scanning mat device was used to objectively evaluate sleep state, which was placed under the bedding, and sleep–wake was judged by comprehensively recording information on respiration, pulse, and body movement detected by a pressure sensor [40,41,64,65]. Similarly, Michael et al. [36] evaluated the sleep state under DHA/EPA using a sleep meter that determines sleep–wakefulness based on body movements and other information, and determined the effects on sleep latency and total sleep time; however, we found no such effects in this study. In the present study, the average latency to fall asleep was approximately 20 min, sleep duration was 371 min, and sleep efficiency was approximately 96%. In the study by Michael et al. [36], the latency to fall asleep was considerably short, averaging 3–4 min, the average sleep duration was 427–455 min, and sleep efficiency was approximately 92%. The differences in participants’ sleep conditions may explain the inconsistent results; however, improved sleep efficiency was observed with DHA/EPA intake in both the studies.

Although no mechanism-related measurements were performed in this study, previous studies have reported that the mechanism by which DHA/EPA significantly improves sleep efficiency involves the regulation of melatonin release and the autonomic nervous system. Regarding the first factor, the pineal gland from which melatonin is secreted has been reported to be affected by DHA/EPA and omega-3 fatty acids. Pineal cells from rats treated with fish oil extract show higher melatonin secretion after noradrenaline or adenosine stimulation [31]. Hamsters fed diets deficient in omega-3 fatty acids had reduced nighttime melatonin levels, and the percentage of DHA in their pineal membrane phospholipids reduced by 76% [32]. DHA has been reported to induce melatonin secretion in mice [66]. Thus, DHA may increase melatonin levels by altering the membrane phospholipid composition of the pineal gland [31,66]. Michael et al. [36] reported no change in the urinary excretion of 6-sulfatoxime, a major metabolite of melatonin in humans after the ingestion of DHA/EPA, but the authors noted that the short duration of urine collection was not sensitive enough to identify an effect of melatonin; therefore, future studies should extend the duration of urine collection, and include measurements of relevant indicators from the blood and saliva to precisely examine the mechanism by which sleep is improved.

The second mechanism involves the regulation of the autonomic nervous system. DHA/EPA increases parasympathetic activity; hence, it is possible that the parasympathetic activity becomes more dominant than the sympathetic activity during sleep, contributing to improved sleep quality. A meta-analysis [67] integrating 15 randomized controlled trials showed by heart rate variability analysis that higher DHA/EPA intake increased parasympathetic nervous system activity.Melatonin is thought to increase sleep quality by making the parasympathetic activity more dominant than the sympathetic activity during sleep [68]. Therefore, DHA/EPA intake may enhance the parasympathetic nervous system and improve sleep quality via melatonin release. As another possible mechanism, Matsumura et al. reported that endogenous PGE2 (prostaglandin E2) in the animal brain is involved in the physiological regulation of sleep–wake activity [69]. EPA has been reported to act on PGE2-producing enzymes and inhibit the formation of PGE2 [70], and it may contribute to improved sleep quality via the inhibition of PGE2 production.

## 5. Conclusions

In this study, we determined that DHA/EPA supplementation can improve sleep quality in middle- and old-aged adults with poor sleep quality. The findings of this study demonstrate that DHA/EPA improves sleep quality in middle- and old-aged adults at doses lower than those administered in previous studies, suggesting that DHA/EPA exerts effects even at low doses. This study serves as a reference for future studies on the efficacy of DHA/EPA on sleep quality.

## Figures and Tables

**Table 1:** Characteristics of participants at baseline.

Variable	DHA/EPA Group (*n* = 33)	Placebo Group (*n* = 33)	*p* Value
*n* (males/females)	11/22	11/22	1.000
Age (years)	52.8 ± 5.9	52.8 ± 5.0	1.000
Weight (kg)	59.8 ± 11.5	58.3 ± 10.5	0.579
Height (cm)	162.6 ± 7.5	163.3 ± 6.4	0.689
BMI (kg/m^2^)	22.5 ± 3.3	21.8 ± 3.3	0.393

**Table 2:** Plasma arachidonic acid, eicosapentaenoic acid, and docosahexaenoic acid levels in the DHA/EPA and placebo treatment groups.

Variable	Baseline			Week 12		
	DHA/EPA Group (*n* = 33)	Placebo Group (*n* = 33)	*p* Value	DHA/EPA Group (*n* = 33)	Placebo Group (*n* = 33)	*p* Value
Arachidonic acid (µg/mL)	228.8 ± 51.3	234.2 ± 57.9	0.687	204.0 ± 59.1	211.6 ± 53.2	0.588
Eicosapentaenoic acid (µg/mL)	52.0 ± 35.3	54.0 ± 38.4	0.824	79.2 ± 40.5 *	56.3 ± 39.1	0.023
Docosahexaenoic acid (µg/mL)	114.0 ± 32.2	117.2 ± 31.3	0.680	137.5 ± 32.6 **	111.0 ± 35.4	0.002

* *p* < 0.05, ** *p* < 0.01.

**Table 3:** Dietary survey for DHA/EPA and placebo treatment groups.

Variable	Baseline			Week 12		
	DHA/EPA Group (*n* = 33)	Placebo Group (*n* = 33)	*p* Value	DHA/EPA Group (*n* = 32)	Placebo Group (*n* = 32)	*p* Value
Energy (kcal)	2391.7 ± 714.6	2261.8 ± 459.5	0.383	2505.9 ± 745.9	2245.7 ± 453.4	0.143
Protein (g)	98.7 ± 35.1	95.6 ± 19.7	0.655	103.5 ± 34.1	95.3 ± 19.3	0.226
Fat (g)	86.2 ± 33.7	84.5 ± 22.2	0.811	90.8 ± 33.6	82.7 ± 20.3	0.165
Carbohydrate (g)	294.3 ± 77.4	268.5 ± 58.2	0.131	307.1 ± 90.4	269.2 ± 55.0	0.172
Omega-3 fatty acid (g)	2.6 ± 1.1	2.5 ± 0.6	0.831	2.7 ± 1.0	2.6 ± 0.7	0.545
Eicosapentaenoic acid (mg)	163.5 ± 97.2	150.9 ± 66.2	0.542	163.2 ± 94.3	164.1 ± 86.1	0.523
Docosahexaenoic acid (mg)	347.1 ± 180.9	337.1 ± 124.0	0.795	352.3 ± 175.7	357.9 ± 147.2	0.659

**Table 4:** OSA-MA score for DHA/EPA and placebo treatment groups.

Variable	Baseline			Week 12		
	DHA/EPA Group (*n* = 33)	Placebo Group (*n* = 33)	*p* Value	DHA/EPA Group (*n* = 33)	Placebo Group (*n* = 33)	*p* Value
Sleepiness on rising	14.3 ± 5.1	14.3 ± 5.1	0.998	17.7 ± 5.4	18.5 ± 5.5	0.488
Initiation and maintenance of sleep	12.9 ± 5.3	13.3 ± 4.3	0.792	16.7 ± 4.9	17.9 ± 5.0	0.351
Frequent dreaming	20.1 ± 8.0	21.0 ± 7.5	0.647	24.4 ± 4.1 *	22.2 ± 6.4	0.043
Refreshing on rising	14.9 ± 3.9	15.0 ± 4.9	0.974	19.2 ± 5.5	20.0 ± 4.8	0.451
Sleep length	16.5 ± 4.7	15.1 ± 4.1	0.194	18.6 ± 4.6	19.2 ± 5.4	0.125

* *p* < 0.05.

**Table 5:** POMS2 scores for DHA/EPA and placebo treatment groups.

Variable	Baseline			Week 12		
	DHA/EPA Group (*n* = 33)	Placebo Group (*n* = 33)	*p* Value	DHA/EPA Group (*n* = 33)	Placebo Group (*n* = 33)	*p* Value
Anger–hostility	46.8 ± 9.0	48.1 ± 12.3	0.955	47.1 ± 10.4	46.9 ± 8.5	0.996
Confusion–bewilderment	47.1 ± 8.9	47.4 ± 8.7	0.633	47.0 ± 9.4	47.5 ± 7.6	0.481
Depression–dejection	49.3 ± 9.5	48.3 ± 8.1	0.889	48.8 ± 8.4	48.0 ± 6.2	0.832
Fatigue–inertia	47.9 ± 8.4	46.7 ± 7.4	0.656	46.2 ± 9.2	46.3 ± 7.7	0.856
Tension–anxiety	50.4 ± 9.0	48.3 ± 9.0	0.537	48.7 ± 9.1	48.8 ± 8.5	0.677
Vigor–activity	52.8 ± 10.7	50.9 ± 9.7	0.356	54.4 ± 10.5	54.5 ± 9.6	0.332
Friendliness	53.0 ± 11.2	52.7 ± 10.0	0.458	53.9 ± 10.9	53.3 ± 9.7	0.414
Total mood disturbance	47.7 ± 8.6	47.6 ± 8.9	0.908	46.8 ± 9.2	46.7 ± 6.9	0.853

**Table 6:** Sleep state test score for DHA/EPA and placebo treatment groups.

Variable	Baseline			Week 12		
	DHA/EPA Group (*n* = 33)	Placebo Group (*n* = 33)	*p* Value	DHA/EPA Group (*n* = 32)	Placebo Group (*n* = 32)	*p* Value
Sleep efficiency (%)	95.6 ± 2.9	96.3 ± 3.1	0.401	96.0 ± 2.6 *	93.7 ± 9.4	0.018
Sleep latency (min)	21.7 ± 12.1	20.9 ± 15.6	0.827	27.8 ± 13.5	21.5 ± 12.6	0.062
Total sleep time (min)	365.9 ± 63.9	384.0 ± 60.9	0.244	378.8 ± 90.4	400.6 ± 89.3	0.867
Actual sleep time (min)	351.7 ± 62.3	370.9 ± 58.4	0.202	364.2 ± 82.4	374.1 ± 68.2	0.468
Average awakening length (min)	14.2 ± 10.5	13.2 ± 12.8	0.715	14.6 ± 12.5	26.6 ± 53.9	0.134
Average ratio of awakening (%)	3.9 ± 2.9	3.2 ± 3.1	0.369	3.5 ± 2.5 *	5.8 ± 9.4	0.018

* *p* < 0.05.

## Data Availability

The data supporting the findings of this study are available from the corresponding author upon reasonable request.

## Supplementary Materials

The following supporting information can be downloaded at https://www.mdpi.com/article/10.3390/nu14194136/s1, Table S1: The results of safety evaluations at baseline and week 12.
