# Supplementary material for: Effect of Docosahexaenoic Acid and Eicosapentaenoic Acid Supplementation on Sleep Quality in Healthy Subjects: A Randomized, Double-Blinded, Placebo-Controlled Trial
Source: Nutrients. 2022 Oct 5;14(19):4136. doi: 10.3390/nu14194136 (PMC9573173; doi:10.3390/nu14194136)
Supplement: Supplementary file 1 [file nutrients-14-04136-s001.zip › nutrients-1932721-supplementary.pdf]

nutrients

MDPI

Supplementary Material

Supplementary Table S1. The results of safety evaluations at Baseline and Week 12.

Variable	Baseline		Week 12	
	DHA/EPA group (*n* = 33)	Placebo group (*n* = 33)	DHA/EPA group (*n* = 33)	Placebo group (*n* = 33)
SBP (mmHg)	115.6±13.7	115.5±12.2	118.9±14.1	119.2±13.6
DBP (mmHg)	73.2±8.9	76.3±10.0	76.9±9.5	77.5±10.5
Pulse rate (bpm)	72.4±11.4	73.6±9.1	73.7±12.6	71.9±9.7
WBC (μL)	5084.8±1428.3	5169.7±1464.5	5033.3±1061.4	4863.6±1111.8
RBC (×10^4/μL)	444.0±39.5	450.5±41.9	451.6±37.6	452.5±48.1
Hb (g/dL)	13.6±1.6	13.9±1.4	13.9±1.6	14.0±1.5
Ht (%)	42.8±4.2	43.5±3.5	43.0±4.2	43.3±4.3
Platelet count (10^4/μL)	27.7±4.0	26.4±5.6	26.4±4.2	25.4±4.9
Total protein (g/dL)	7.0±0.4	7.1±0.4	7.0±0.4	7.1±0.3
Total bilirubin (mg/dL)	0.78±0.29	0.87±0.32	0.85±0.31	0.89±0.30
AST (U/L)	19.8±4.4	23.5±9.1	24.9±24.2	22.6±6.0
ALT (U/L)	17.9±7.0	21.6±14.5	21.5±17.2	20.2±11.0
LDH (U/L)	176.6±25.4	184.2±28.0	183.8±29.8	184.2±28.9
ALP (U/L)	190.1±57.1	181.5±52.3	185.1±55.0	173.0±47.0
γGTP (U/L)	27.1±19.2	31.6±25.8	29.4±22.4	30.8±25.5
BUN (mg/dL)	12.6±2.8	13.0±3.3	13.4±2.9	13.0±2.7
Creatinine (mg/dL)	0.72±0.11	0.71±0.15	0.72±0.12	0.71±0.15
Na (mEq/L)	140.3±1.6	13.9±1.4	13.9±1.6	14.0±1.5
Cl (mEq/L)	101.6±2.0	100.8±2.2	101.7±3.0	101.3±1.5
K (mEq/L)	3.9±0.3	3.9±0.3	4.2±0.4	4.2±0.3
Total cholesterol (mg/dL)	222.9±33.1	228.2±33.1	235.5±37.8	237.4±36.0
LDL-cholesterol (mg/dL)	131.2±34.1	135.9±27.2	137.9±36.8	139.7±29.4
HDL-cholesterol (mg/dL)	76.5±19.2	77.6±22.6	80.2±20.5	78.9±22.9
TG (mg/dL)	85.8±55.6	91.5±53.5	73.2±32.3	91.8±55.1
FPG (mg/dL)	88.4±10.4	85.4±7.7	88.3±8.8	85.3±7.4
HbA1c (%)	5.4±0.3	5.5±0.3	5.4±0.3	5.4±0.2

Data are represented as mean ± SD ; body mass index (BMI), systolic blood pressure (SBP), diastolic blood pressure (DBP), white blood cell (WBC) count, red blood cell (RBC) count, hemoglobin (Hb), hematocrit (Ht), aspartate aminotransferase (AST), alanine aminotransferase (ALT), lactate dehydrogenase (LDH), alkaline phosphatase (ALP), γ-glutamyl trans-peptidase (γGTP), blood urea nitrogen (BUN), sodium (Na), chlorine (Cl), potassium (K), triglyceride (TG), fasting plasma glucose concentration (FPG), and glycated hemoglobin (HbA1c).
